# Microwave Ablation of Primary Malignant Pelvic Bone Tumors

**DOI:** 10.3389/fsurg.2019.00005

**Published:** 2019-03-05

**Authors:** Qing-Yu Fan, Yong Zhou, Minghua Zhang, Baoan Ma, Tongtao Yang, Hua Long, Zhe Yu, Zhao Li

**Affiliations:** Department of Orthopedics, Tangdu Hospital, Fourth Military Medical University, Xi'An, China

**Keywords:** malignant pelvic bone-tumor, microwave, ablation, limb-salvage, thermotherapy

## Abstract

**Background:** En bloc tumor resection followed by reconstruction is a widely used surgical treatment for malignant pelvic bone tumors. High rates of complications and mechanical instability often contribute to poor postoperative results. We attempted en bloc microwave ablation (MWA) *in situ* to improve the outcome.

**Methods:** From May 1995 to December 2015, 104 patients with primary pelvic malignancy received radical MWA in our department. After careful dissection of the tumor-bearing bone from surrounding normal tissues with safe margins, a microwave antenna array was inserted into the tumor mass to emit electromagnetic energy, inducing tumor cellular death via thermocoagulation. The loose, devitalized tumor tissues were removed by cutting or curettage, leaving a defective bone scaffold. Re-strengthening by autograft or allograft was needed in most patients.

**Results:** The over 3 years survival rate was 51.5% for high-grade malignancies (among them, 26.9% were osteosarcoma) and 94.8% for low-grade malignancies (chondrosarcoma). In most of the living patients, cosmetic and useful limbs were preserved. The mean functional score (Musculoskeletal Tumor Society) was 27 or 90% (range: 25–30, 75–100%). Among the 56 patients who belonged to the excellent function group, 11 were followed up for more than 10 years. The local recurrence rate was 8.6%. Among the 9 patients with recurrence, 5 died from disease, 2 were treated by hemipelvic amputation, and 2 underwent revision surgery with MWA and gained local control. The deep infection rate was 5.6%. All six patients with infection were healed by irrigation, debridement, and systemic antibiotic administration.

**Conclusion:** Local, microwave-induced hyperthermia for treating malignant pelvic bone tumors is an effective alternative method. The oncological and functional results are encouraging. The use of MWA should be continued to evaluate and improve this new therapeutic system.

## Introduction

**Figure 1:**
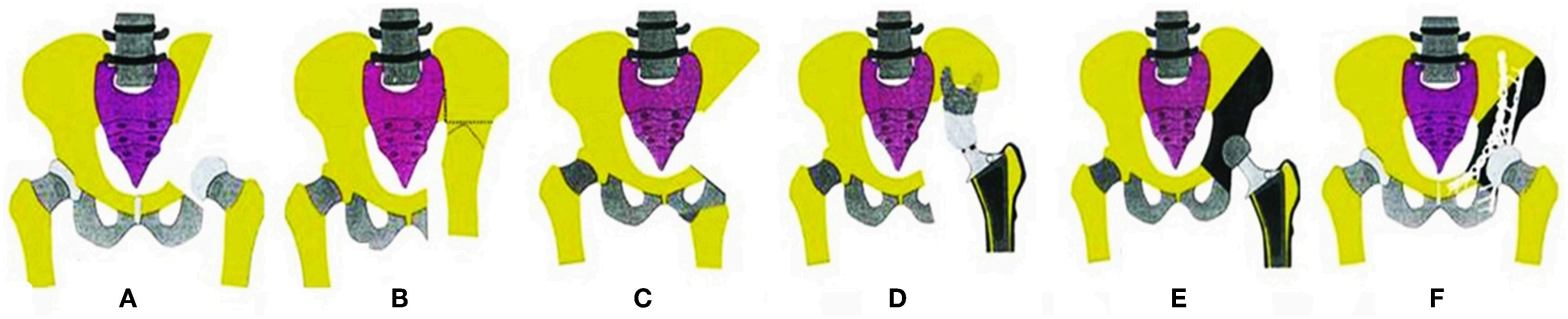
Schematics show the reconstruction procedures after internal hemipelvectomy. **(A)** No reconstruction, leaving a flail hip joint. **(B)** Ilio-femoral fusion. **(C)** Ischial-femoral fusion. **(D)** Saddle prosthesis replacement. **(E)** Designed special endoprosthesis or 3-D printed prosthesis replacement. **(F)** Pelvic allograft or extracorporeal irradiation and re-implantation after en bloc resection.

The pelvic girdle is a common location for primary bone sarcomas and metastatic tumors, with the periacetabular region being the most frequent site. For the majority of cases, surgical resection (in addition to chemotherapy) remains the only hope of a cure (1, 2). Because of the complicated anatomy and depth of the region, the tumors are often large when the final diagnosis is made, and performing a complete surgical resection becomes difficult. During surgery, the proper margins are difficult or impossible to obtain for cases that are diagnosed late even though they are essential for a positive final outcome. Internal hemipelvectomy together with resection of the hip joint usually results in a large defect that should be reconstructed to restore the bony anatomy and mechanical stability of the pelvis. Otherwise, it will result in a frail or short extremity. The reconstruction procedures include mega-endoprosthetic replacement, biological reconstruction using autografts or allografts, and hip transposition. The schematic shows the main reconstruction types (3) (Figure 1).

Unfortunately, the aforementioned procedures have a significant risk of negatively influencing functional outcome (4, 5). Furthermore, there is a high rate of complications, such as recurrence (ranging from 21 to 38%) and infection (ranging from 20 to 47%), which seems inherent with the magnitude of the surgery (6–10). Once a deep infection occurs, the pelvic reconstruction devices often have to be removed. Prosthesis loosening and mechanical instability make the functional results worse. Patients with pelvic malignancy face an increased risk of treatment failure (11). For pelvic tumors, limb salvage surgery is still a great challenge, even to the most experienced orthopedic oncologists.

From July 1995 to December 2015, 104 patients with primary malignant pelvic bone tumors were treated in our institute by microwave ablation (MWA) as a definitive measure for limb salvage. The results are presented here.

## Materials and Methods

This is a retrospective study approved by the Human Subjects' Committee of Tangdu Hospital, the Fourth Military Medical University of China. One hundred and four patients with high- or low-grade primary malignant tumors of the pelvis were treated by MWA. Chondrosarcoma, osteosarcoma, and Ewing's sarcoma were the most common diagnoses. Among all of the cases, 66 were classified as high-grade malignancies, and 38 were low-grade malignancies. The present series is composed of cases with available follow-up data (Table 1).

**Table 1:** Clinical data of 104 patients with primary malignant pelvic bone tumors.

**Classification**	**Total number**	**Gender**	**Average age**
Chondrosarcoma	38	Male: 26; Female:12	39
Osteogenic sarcoma	26	Male: 18; Female: 8	24
Ewing's sarcoma	11	Male: 5; Female: 6	22
Malignant fibrous histiocytoma	7	Male: 3; Female: 4	47
Lymphoma	5	All male	31
Synovial sarcoma	3	Male: 2; Female: 1	42
Malignant hemangioma	3	All female	42
Leiomyosarcoma	2	All male	33
Myeloma	2	All male	53
Malignant neurofibroma	2	Male: 1; Female: 1	38
Mesenchymal sarcoma	1	Female	33
Fibrosarcoma	1	Female	22
Spindle sarcoma	1	Male	37
Clear cell sarcoma	1	Male	75
Reticular cell sarcoma	1	Male	44

**Figure 2:**
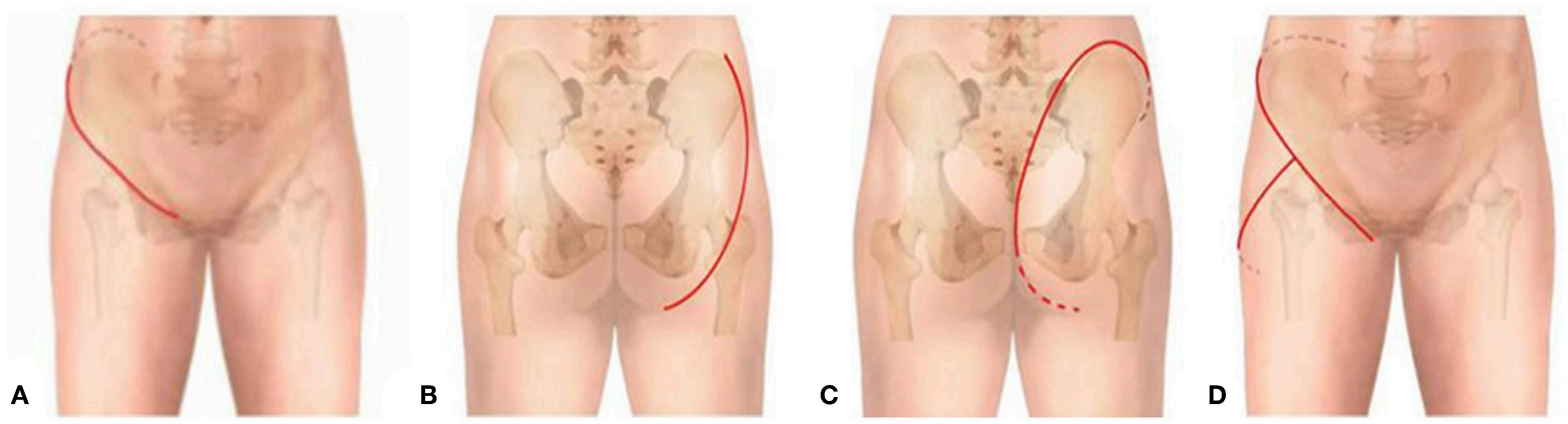
Schematic of incision options. **(A)** Anterior approach. **(B)** Posterior approach 1; the myocutaneous flap of the gluteus maximus was retracted medially. **(C)** Posterior approach 2; the myocutaneous flap of the gluteus maximus was retracted laterally. **(D)** “T” type approach.

To generate a careful preoperative plan, detailed imaging data were necessary to determine the bony and soft-tissue range of the tumors and their relation to vital structures, such as major blood vessels, nerves, and the pelvic viscera. All patients diagnosed with an osteosarcoma or Ewing's sarcoma underwent the appropriate protocols for neoadjuvant chemotherapy. All patients underwent surgery under general anesthesia. The operations were generally carried out with the patients in a lateral position. The incision was chosen by the involvement of the tumor: if the tumor mainly involved the ischium, a posterior exposure was performed; if the tumor mainly involved the pubis, the retroperitoneal space was entered through the ilioinguinal approach. For tumors involving the II region or iliosacral joint, both intrapelvic and extrapelvic exposures were needed through a “T” type incision (Figure 2).

**Figure 3:**
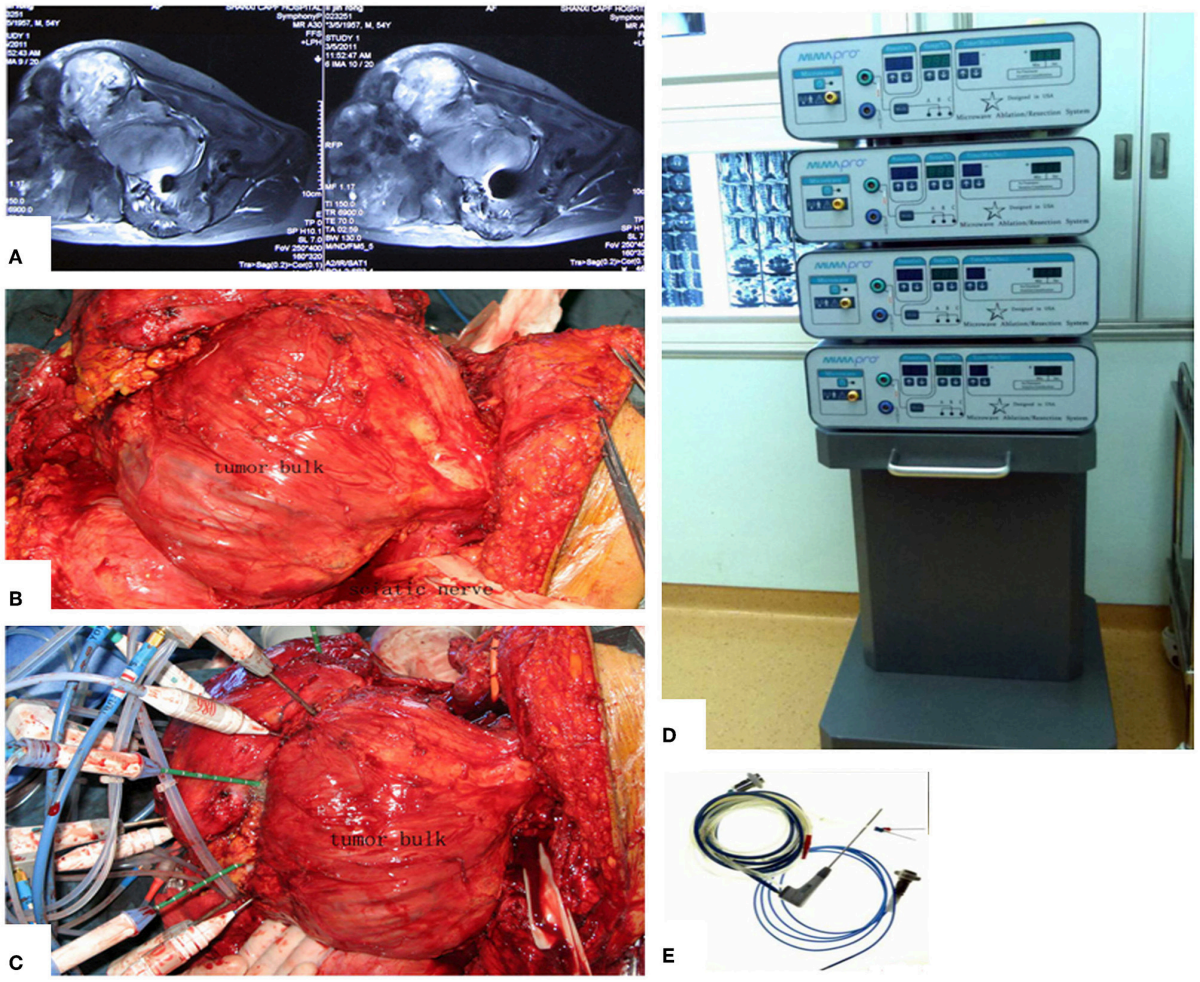
MWA process and microwave generator. **(A)** MRI shows a massive pelvic malignancy. **(B)** Exposure and dissection of the tumor mass from surrounding normal tissues. **(C)** Insertion of the antenna and thermocouples into the tumor mass, usually at the junctional zone of the tumor bulk and ilium bone. **(D)** Microwave generator. **(E)** Antenna.

**Figure 4:**
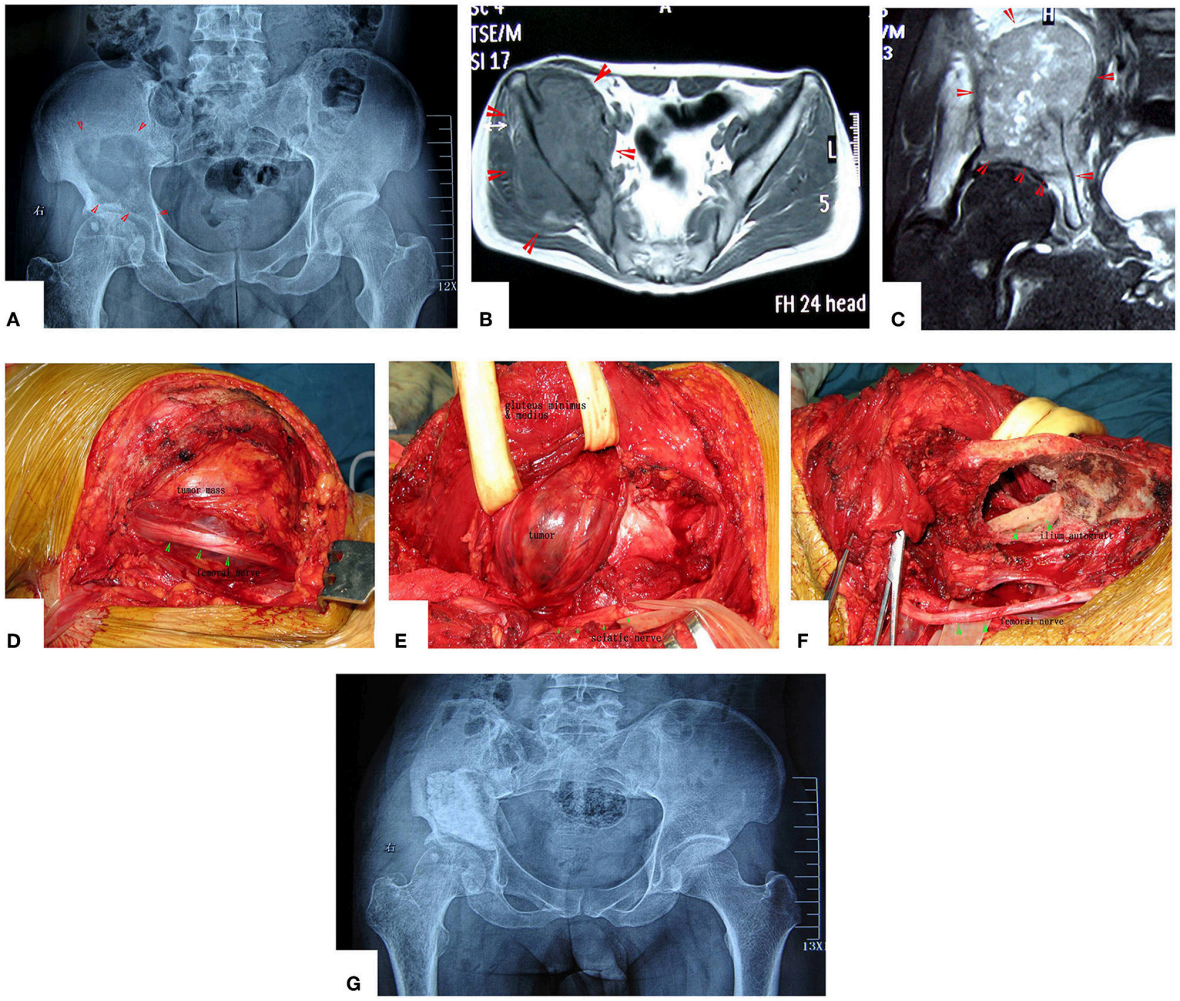
A patient with pelvic MHF treated by MWA. **(A)** X-ray before surgery shows osteolytic destruction of the cortical bone and extraosseous tumor expansion. **(B,C)** MRI shows a massive tumor very close to the hip joint. Notably, the hip joint is not infiltrated. **(D)** Internal pelvic space exposure; notice the compressed femoral nerve. **(E)** The loose, devitalized tumor tissues were removed after MWA. **(F)** The re-strengthening procedure included using an autograft from the ilium and a mixture of cement and morselized allograft bone chips. **(G)** X-ray after the operation.

**Figure 5:**
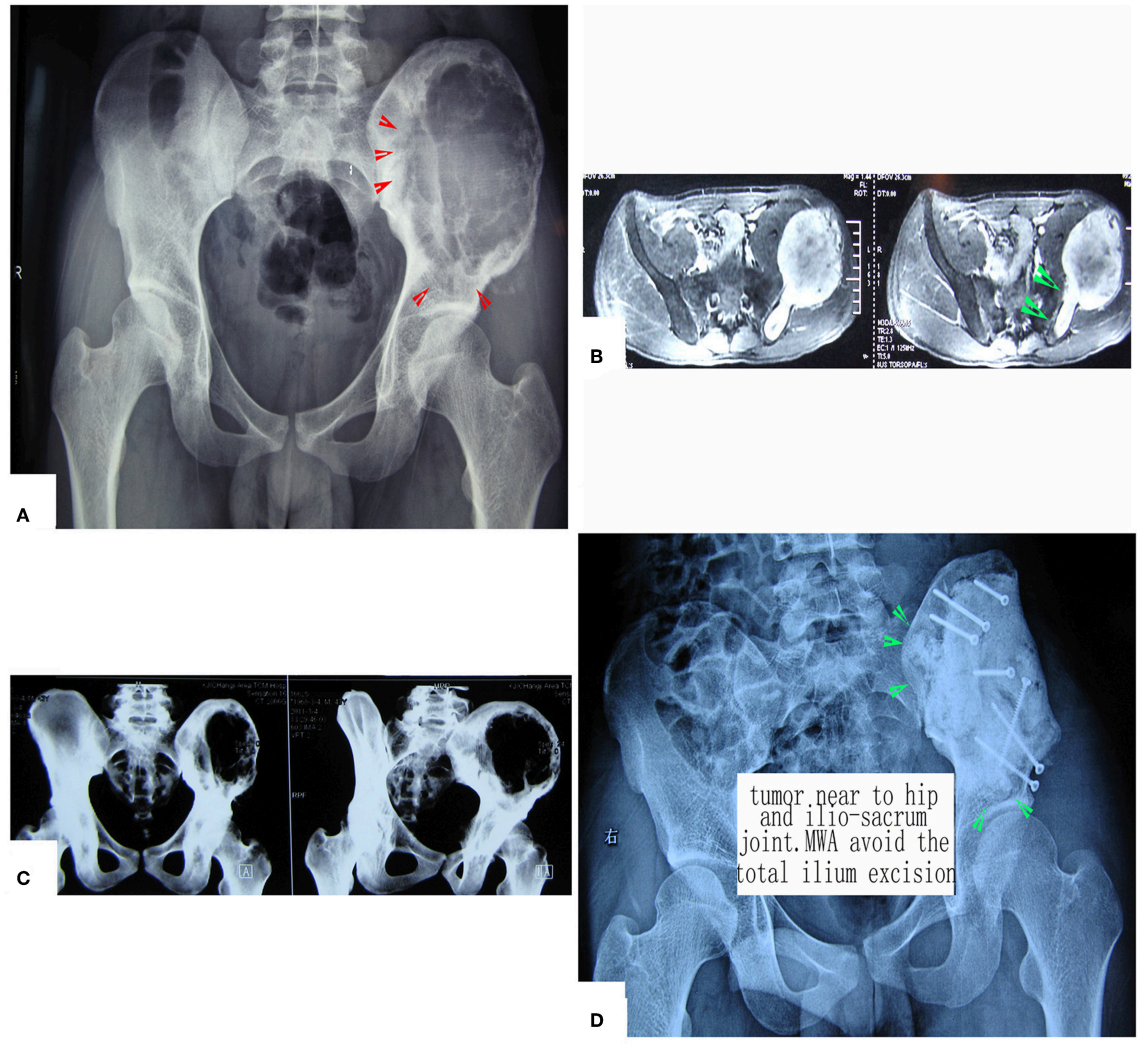
Treatment of a patient with pelvic mesenchymal sarcoma close to the hip joint. **(A–C)** X-ray and CT scan. Images show a large osteolytic lesion near the hip joint. **(D)** X-ray after the operation. After MWA, the defect was reconstructed by long screws into the residual ilium bone, and a mixture of cement and morselized allograft bone chips was used to make an artificial pelvic “wall.” The hip joint was reserved completely. The patient was followed up for 7 years, and his hip function is pretty good.

**Figure 6:**
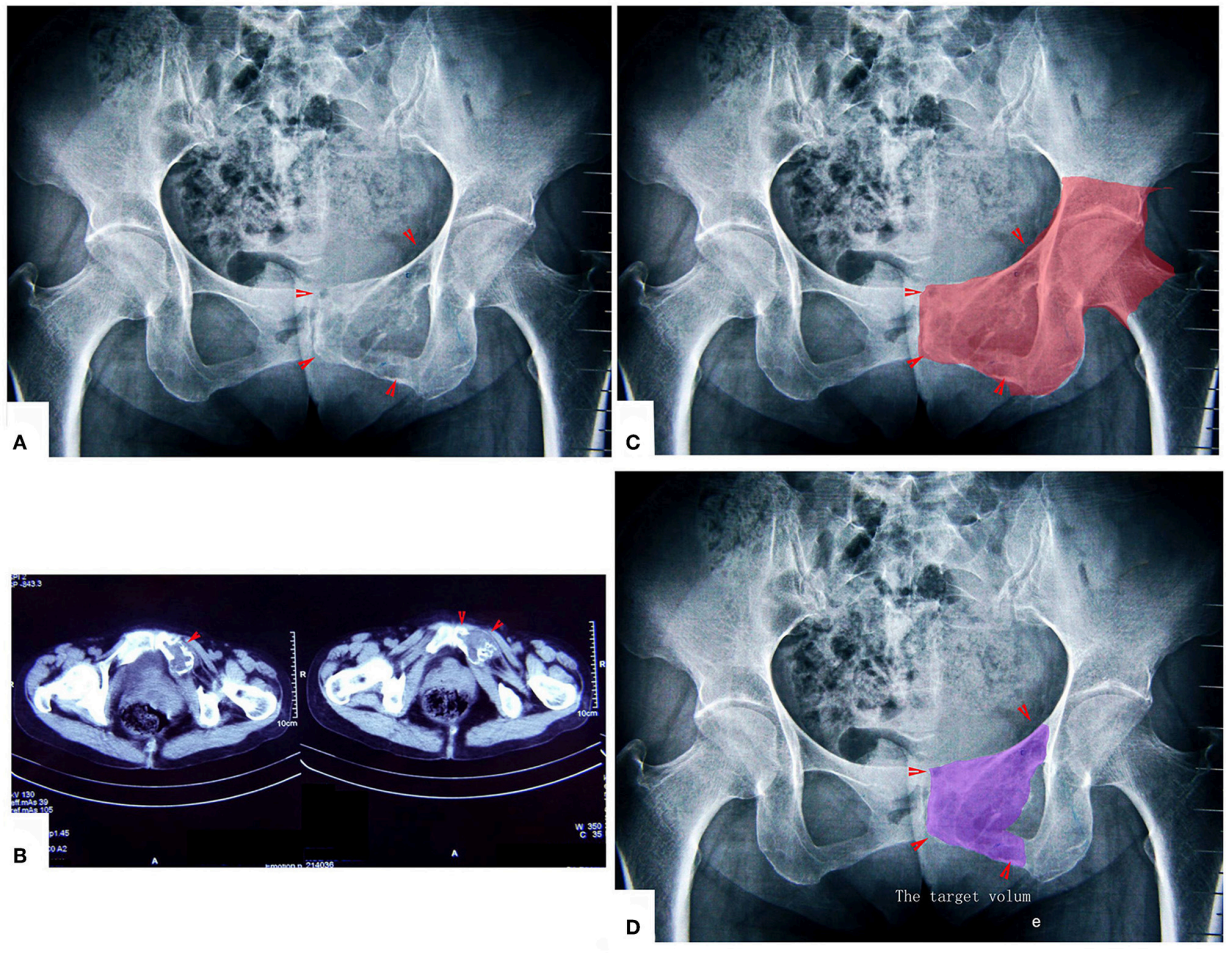
A patient with chondrosarcoma of the pecten pubis and inferior pubic ramus. **(A,B)** X-ray and CT scan. The lesion was near the inner wall of the hip joint. **(C)** The colored region shows the range of traditional treating method (en bloc resection). **(D)** The colored region shows the target volume for MWA treatment.

**Figure 7:**
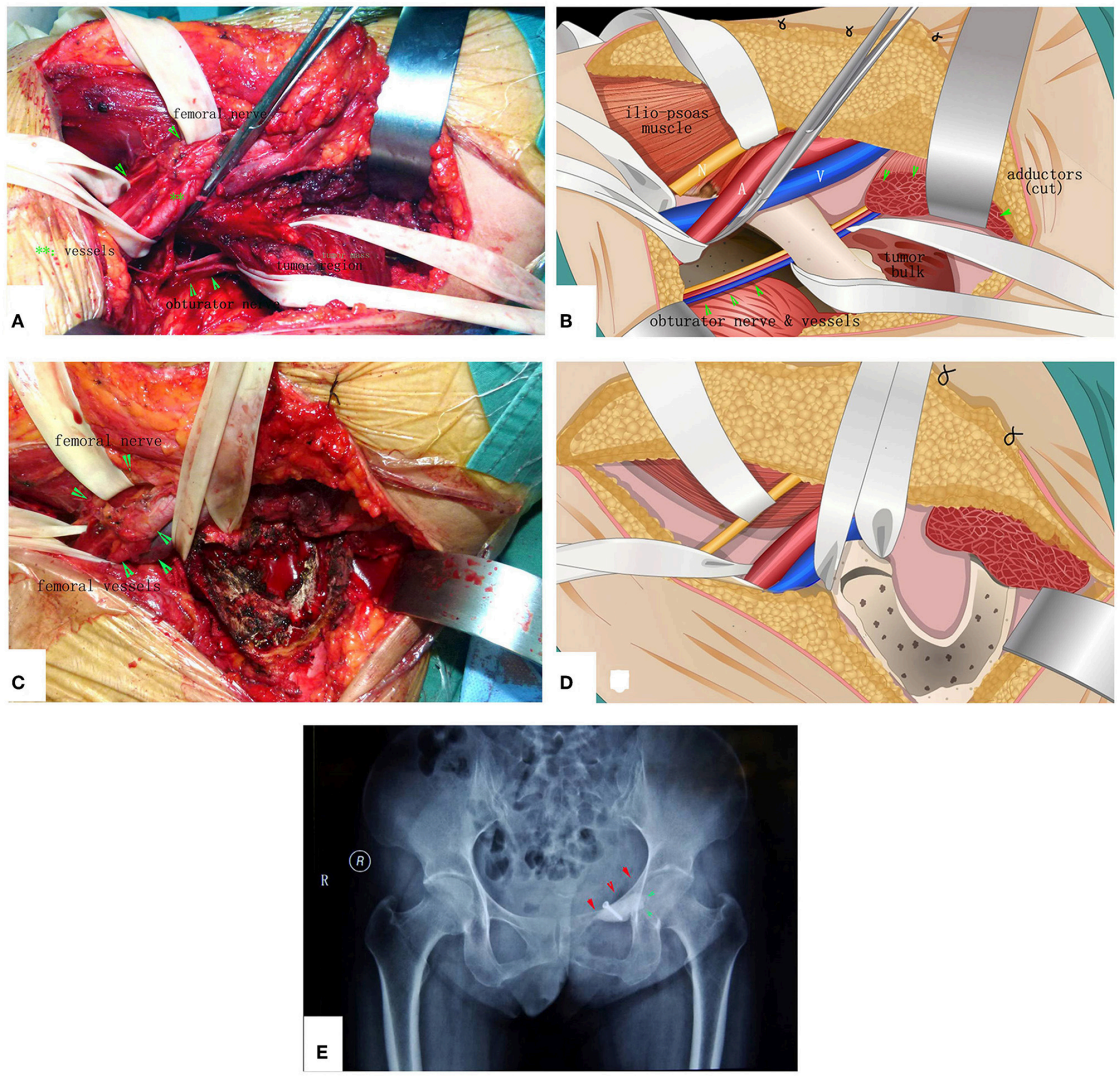
The surgical procedure of the patient shown in Figure 6. The obturator neurovascular bundle was protected. **(A)** Exposure. **(B)** The schematic of **A**. **(C)** After MWA and curettage. **(D)** The schematic of (**C**). **(E)** X-ray after the operation.

**Figure 8:**
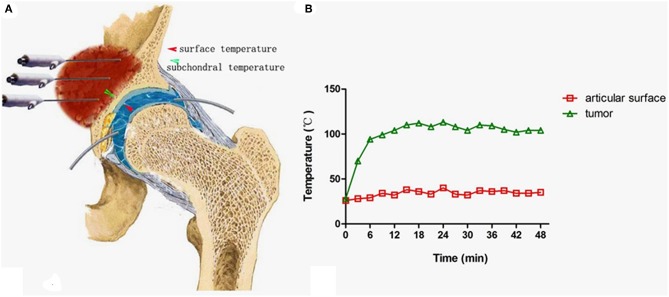
Measures to protect the joint cartilage from overheating when the tumor is very close to the joint surface. **(A)** A tube was inserted into the space between the femoral head and acetabulum for infusion of cooled saline to protect the cartilage (Note: the negative pressure should be overcome by pulling the leg until a clear sound such as a “pop” can be heard.). **(B)** The temperature curve at both sides of the acetabulum cartilage.

**Figure 9:**
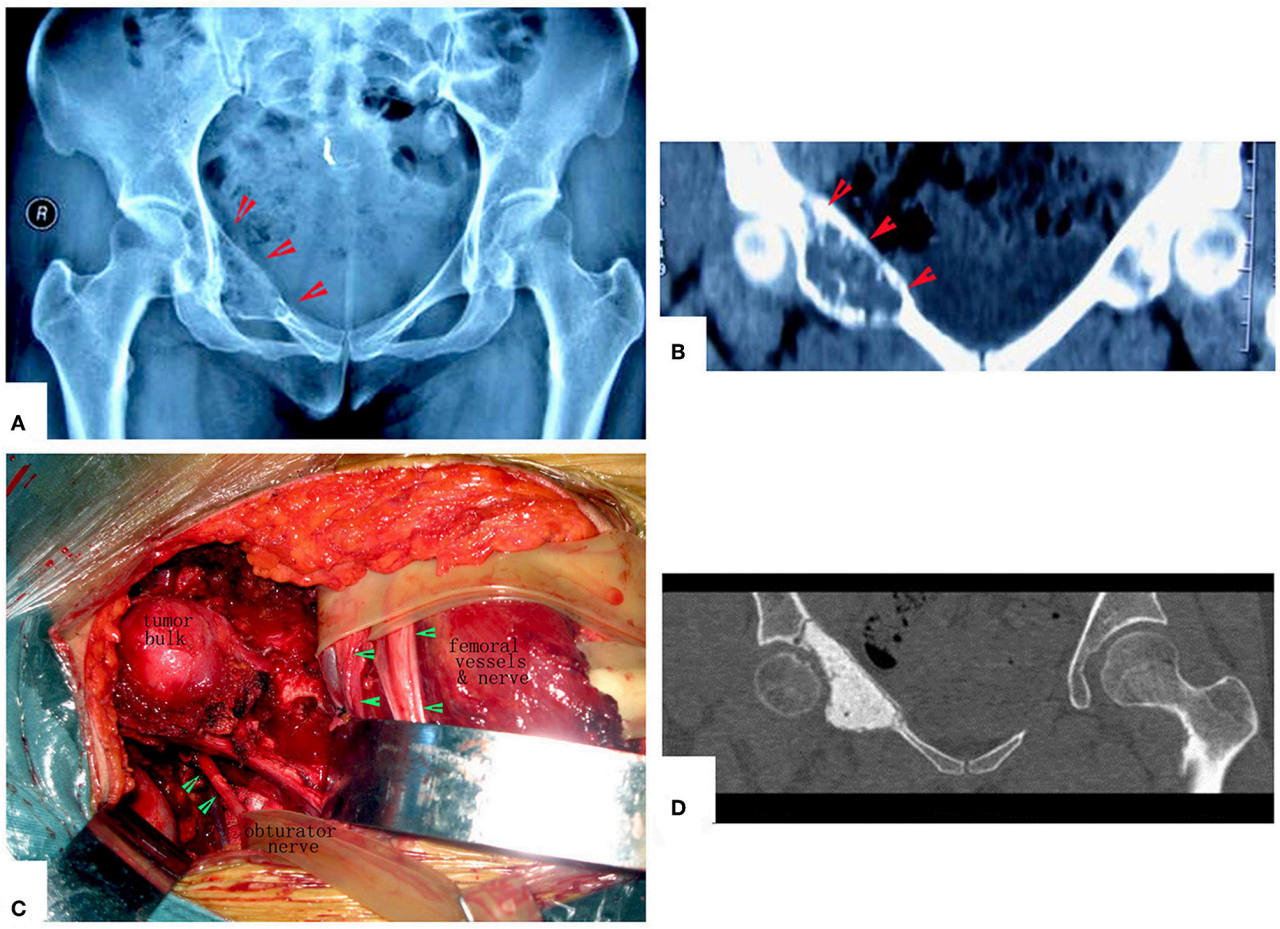
Management of periacetabular chondrosarcoma. **(A,B)** X-ray film and CT scan. Anteroposterior radiograph of the pelvis showing a periacetabular chondrosarcoma on the right. CT of the pelvis, showing the destruction of the inner part of the upper pubic bone. **(C)** Exposure of the tumor. **(D)** Images after the operation. The hip joint reserved well. The function is perfect at 8 years after operation.

**Figure 10:**
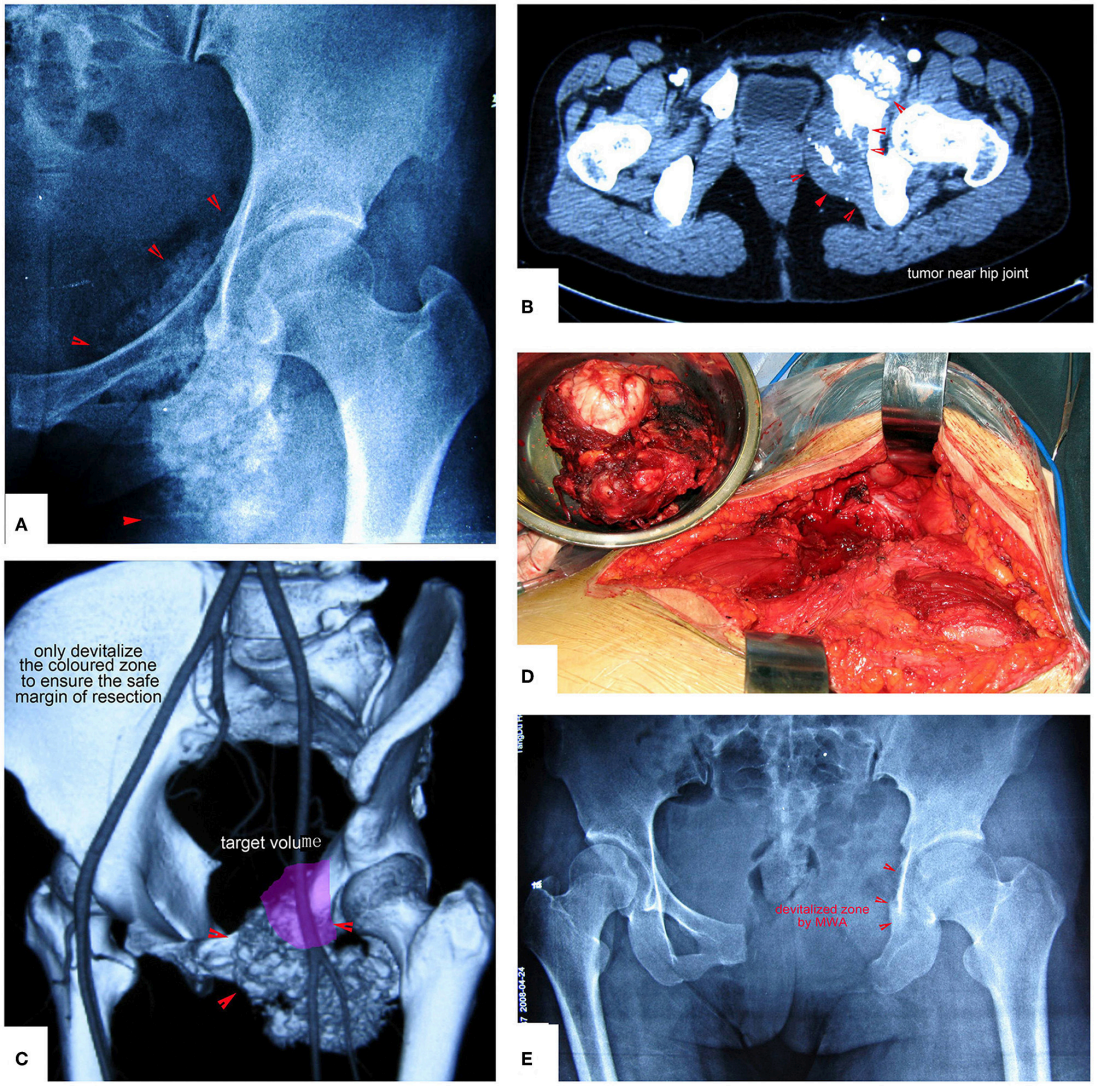
Chondrosarcoma of the pubis and ischium near the hip joint treated by resection plus MWA. Only the colored zone was devitalized to ensure a safe margin of resection. **(A,B)** Images show the tumor near the hip joint. Simple resection of the pubis and ischium cannot ensure a safe margin. **(C)** The colored region is the target volume for MWA. **(D)** The resected tumor specimen. **(E)** X-ray after the operation.

**Figure 11:**
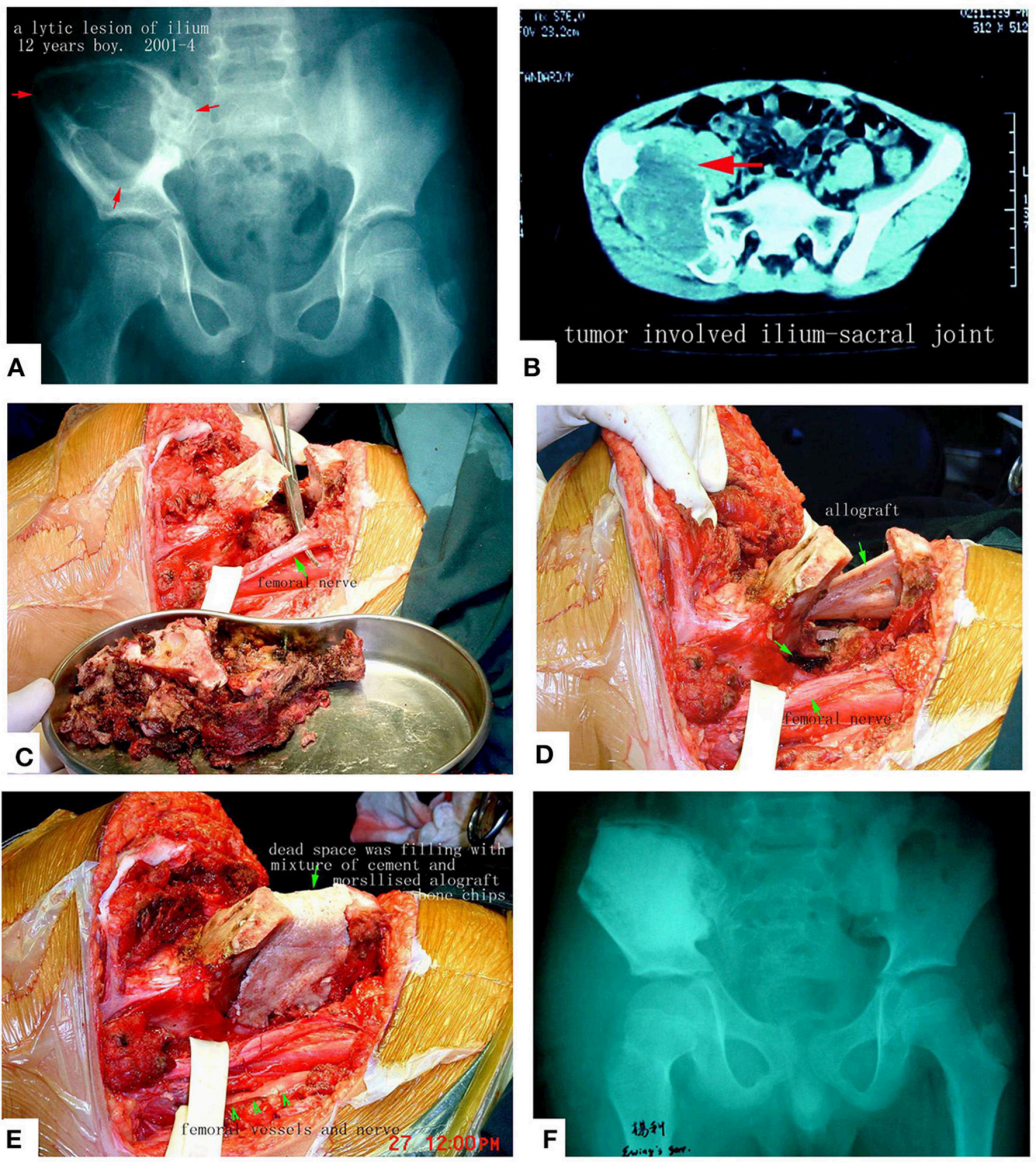
Pelvic Ewing's sarcoma treated by MWA. **(A)** X-ray film before surgery. **(B)** CT scan. **(C)** The soften, devitalized tumor tissues were removed. **(D,E)** Reconstruction by allograft and cement. **(F)** X-ray after the operation. The patient's function is perfect.

**Figure 12:**
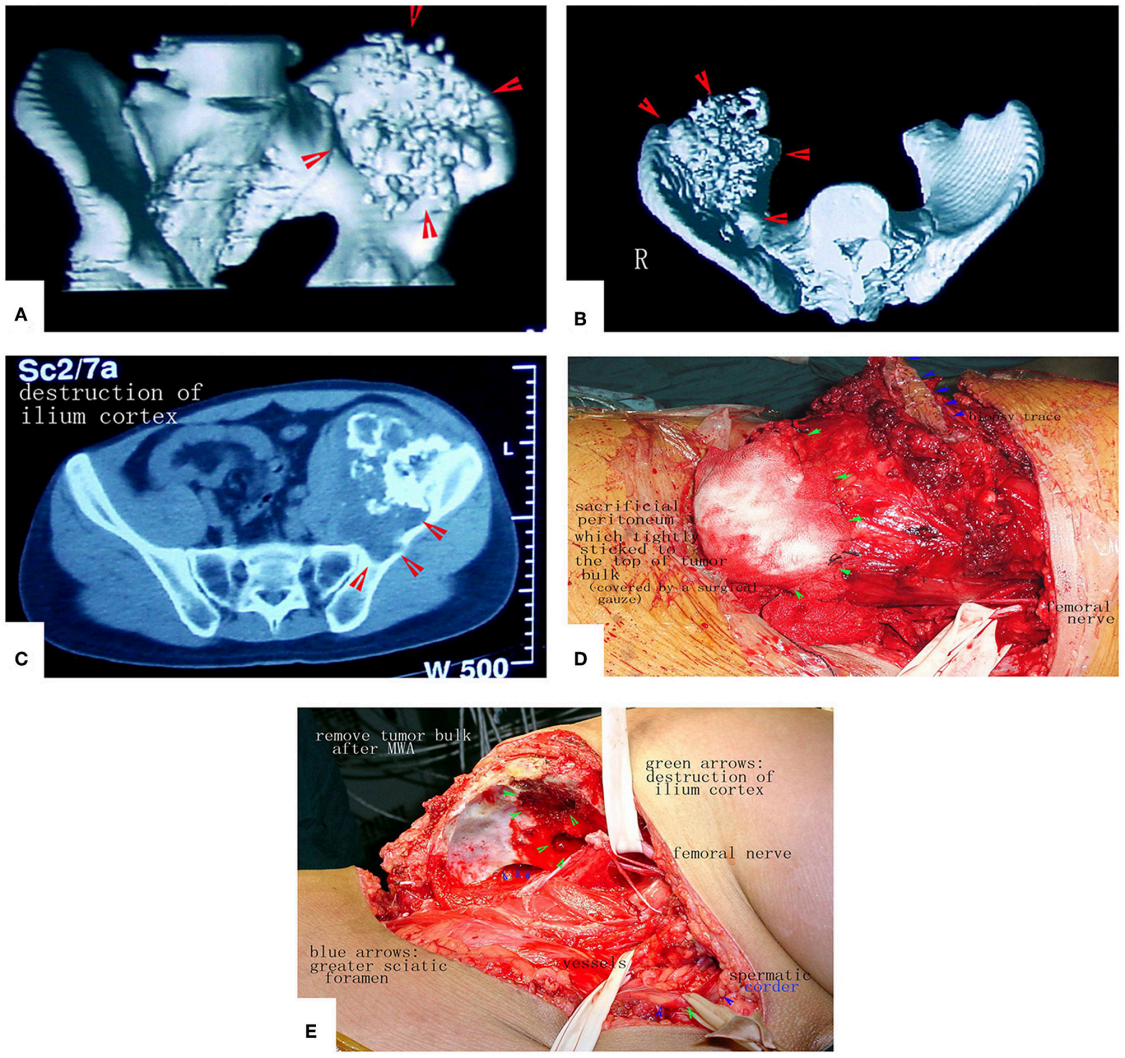
A patient with a massive pelvic chondrosarcoma was treated by MWA. **(A–C)** Images of the tumor. CT scan showed huge tumor of pelvis. **(D)** Due to the strict adhesion between the peritoneum and tumor bulk, a portion of the peritoneum that adhered tightly was sacrificed to prevent tumor cell contamination. **(E)** The tumor mass was removed. No reconstruction was needed.

**Figure 13:**
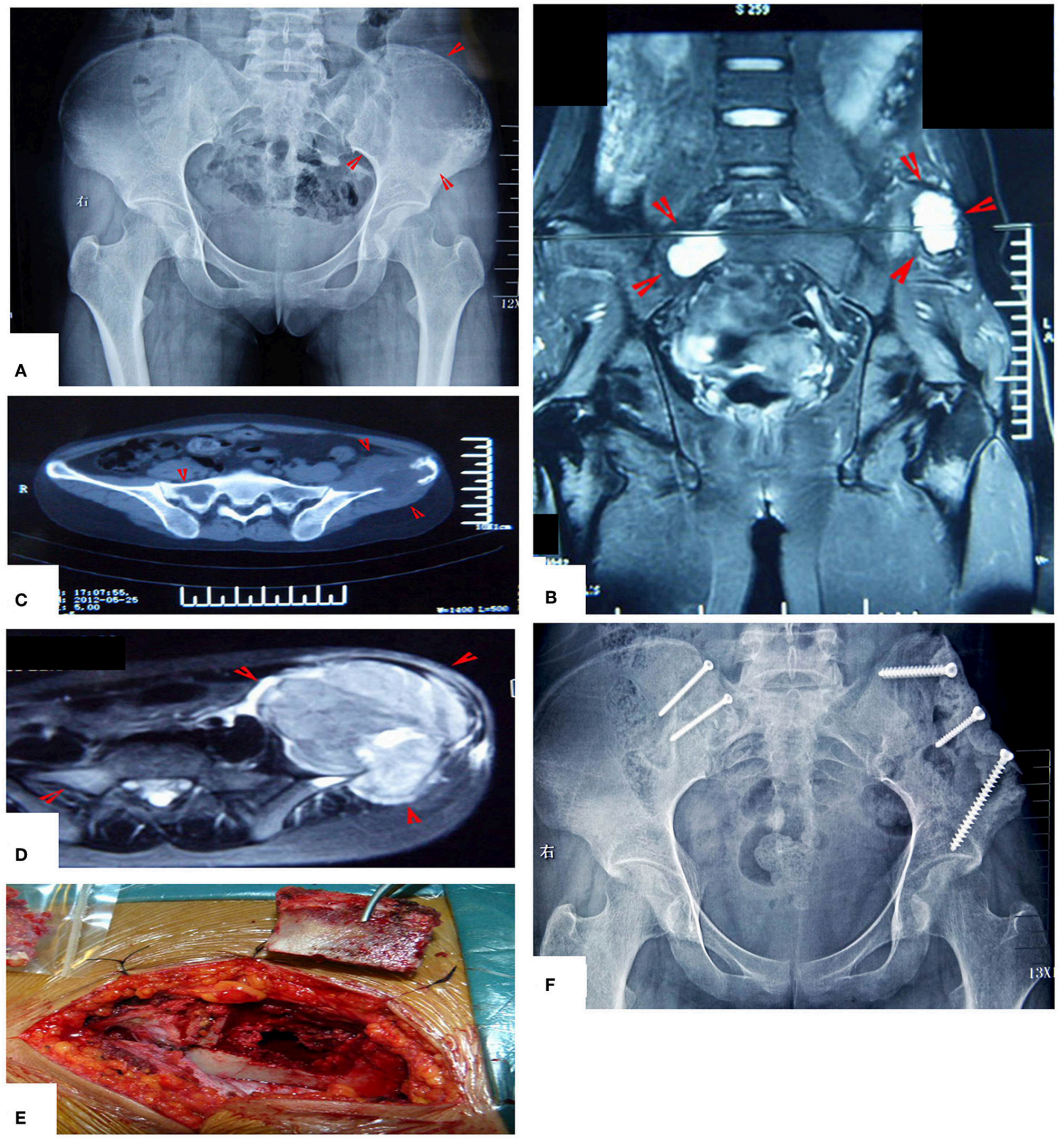
MWA treatment of a patient with bilateral Ewing's sarcoma. **(A)** X-ray film before operation. **(B–D)** MRI images show the bilateral lesions. **(E)** The lesion on the left side was treated as described above. The lesion on the right side was treated through a bone window and the bone bloc was fixed *in situ* by two screws after MWA. **(F)** X-ray after the operation.

**Figure 14:**
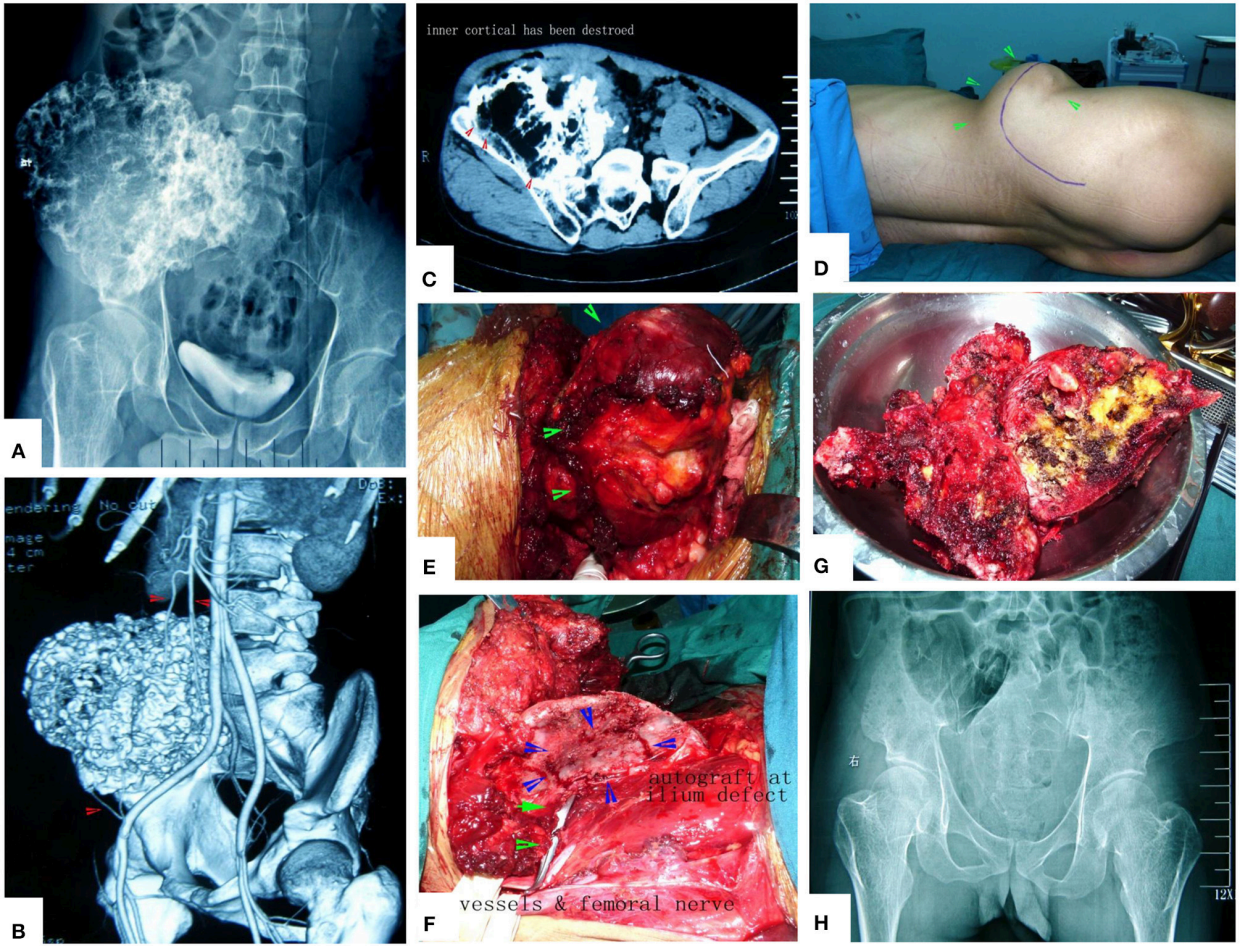
A case of huge pelvic peripheral chondrosarcoma. The surgical procedure was greatly simplified by MWA. **(A)** X-ray film before operation. **(B)** Digital angiography. **(C)** CT scan before surgery. **(D)** Incision. **(E)** The tumor bulk has been separated from surrounding normal tissues. **(F)** After MWA and reconstruction. **(G)** The specimen. **(H)** X-ray film after operation.

The main aim of the preoperative planning was to determine the target volume. According to Goldberg S Hahum, thermal ablation is performed to elevate the temperature of the target volume to 50–100°C (12). The procedure involves the exposure and dissection of the tumor-bearing bone and its extraosseous mass from the surrounding normal tissues, including the vital structures such as femoral nerve, sciatic nerve, and external iliac vessels, as well as the ureter, bladder, and rectum if the tumor has extended into the ischioanal fossa. Usually, these structures can be dissected away from tumor bulk due to the presence of a pseudocapsule. After completion of the intrapelvic and/or extrapelvic dissection of the soft tissues with a proper margin, two or more pieces of surgical gauze were passed through the great sciatic foramen to cover the nerve as a measure to decrease possible overheating of the nerve by applying cool saline solution dropwise to the gauze. Several other pieces of gauze were placed between the normal tissues and tumor bulk. The antenna array consisting of 3–8 antennae was evenly inserted into the tumor according to the tumor size. The distance between two antennae was <3 cm. Three or four thermocouples were placed in various critical locations to monitor the temperature within and around the tumor bulk. The duration of microwave irradiation was also dependent on the tumor size. The aim was to ensure that the temperature at ***any part*** of the tumor-bearing bone (so called target volume) reached 80°C or higher and was maintained for at least 20–30 min. After MWA was accomplished, the loose, devitalized tumor tissues were removed by cutting or curettage, leaving behind the defective bone as a scaffold for reconstruction. The continuity of the sciatic notch was preserved in all but three cases. A re-strengthening procedure was needed in the majority of patients with autografts or allografts. A series of figures illustrate the surgical procedure at different anatomical sides (Figures 3–14).

Weight-bearing activity was not allowed for 6 weeks before complete soft-tissue healing.

## Results

Follow-up range: 3–20 years.

### Oncological Results

1. Among 66 patients with high-grade malignant tumors, 32 patients died from the lesion; the remaining 34 patients developed neither metastasis nor local recurrence after 3–20 years, and the over 3 years survival rate was 51.5%. The patients with osteosarcoma had only a 26.9% over 3 years survival rate (7/26). The patients with Ewing's sarcoma had a good prognosis, with an over 3 years survival rate that reached 90.9%.
2. Among 38 patients with low-grade malignant tumors (chondrosarcoma), 2 died from disease, and the over 3 years survival rate was 94.8%.

### Functional Results

Simple and succinct criteria proposed by Aboulafia and Malawer were used for grading results (10). The overall results were graded according to 3 criteria: meeting the oncological goal of surgery to achieve local control; possessing the ability to be a community ambulator without pain, with or without support aids; and meeting the requirement for analgesia.

Thirty-seven patients who died or lived with disease were excluded from the function analysis. Among the remaining 67 patients with high- or low-grade malignancy, 53 had excellent hip function, were stable, were painless, and had almost full range of motion; however, 24 patients had a mild Trendelenburg gait because the gluteus minimus or/and gluteus medius had to be sacrificed. These patients still can be categorized as having an excellent result according to the standards. Among the 53 patients who belong to the excellent function group, 11 were followed up for more than 10 years. Nine patients had good hip function, and 5 had poor hip function. The functional score (Musculoskeletal Tumor Society) varied from 25 to 30 (75 to 100%), with a mean score of 27 (90%).

### Complications

Local recurrence occurred in 9 patients (8.6% local recurrence rate); 2 patients were treated by hemipelvic amputation, 2 accepted revision surgery using MWA and were followed up for more than 3 years, and another 5 died from lesions. Six patients developed a deep infection (5.6%), which was resolved by irrigation, debridement, and administration of systemic antibiotics. A fistula developed in one patient. One incurred lumbosacral trunk damage during heating because the nerve could not be separated from the tumor. Two patients sustained sciatic notch fractures, which healed in a non-anatomical position. Four patients suffered hip joint degenerative changes.

## Discussion

Cellular homeostasis can be maintained with a mild elevation of temperature to ~40°C. When temperatures are increased to 46°C for 60 min, irreversible cellular damage occurs. Between 60–100°C, there is near instantaneous induction of protein coagulation, which irreversibly damages key cytosolic and mitochondrial enzymes, as well as nucleic acid-histone protein complexes (13). The main concept of the novel MWA method is to achieve tumor en bloc ablation *in situ* with safe margins (assuming that the tumor is localized in the region of diagnosis) using antenna-guided hyperthermia therapy.

Reports concerning the use of increased temperatures (hyperthermia) in the treatment of malignancies have existed for many centuries (14). A large number of basic studies have revealed that tumor cells are more sensitive to hyperthermia than normal cells (14–16).

Electromagnetic microwaves can be used to heat the tumor mass by agitating polar water molecules in the tumor tissue. The friction of water molecules produces heat, thus inducing cellular death via coagulation necrosis. The increase in intratumoral temperature was fast during ablation of large tumor volumes with multiple antenna arrays. The ablation effect of hyperthermia depends on the temperature and exposure time. Our study data show that heating to 60–80°C or higher for 20–30 min is guaranteed to kill all of the living cells while the inductivity capacity (new bone regeneration) and conductivity capacity (support for body weight) of the heating-necrotizing bone frame can be partially maintained (13).

Local tumor control depends on the adequacy of the surgical procedure or, in other words, on the proper margin gained by resection. If a traditionally defined wide margin can be identified using the en bloc resection technique, MWA can achieve a similar goal while retaining a curetted cortical bone scaffold, thus making reconstruction easier and more durable.

The prognosis of pelvic osteosarcoma is very poor because the lesion develops quickly, and a large tumoral thrombus can be found (in this series, 3 patients had a thrombus, as shown in Figure 14). Therefore, early diagnosis is important.

MWA is a simpler procedure than traditional en bloc resection followed by reconstruction. The limb-saving surgery for pelvic malignancy can be divided into three steps: the first step involves dissection and exposure, the second step involves resection or ablation, and the third step involves reconstruction. This surgery is similar to the traditional resection and MWA procedures at the first step. At the second step, MWA needs more time than resection, but almost no bleeding occurs during MWA, while there are varying amounts of bleeding during en bloc resection. At the third step, traditional reconstruction requires more time than MWA, and sometimes, the patients who receive MWA do not need any kind of reconstruction.

The novel method reported herein has the following advantages. (1) En bloc MWA greatly improves the functional outcome and durability because it can maintain leg length and pelvic girdle stability. Furthermore, the devitalized bone frame is similar to a biologically autogenous graft so that when healing does occur, it is permanent and does not have the potential for loosening, which is inherent with a metal prosthesis. (2) MWA greatly simplifies the surgery process, shortens the operation time and decreases the early and late complication rates. (3) MWA is a cheaper operation than prosthesis replacement (3-D printed prostheses are very expensive).

Our IRB did not find the results to be inferior early in the study, and therefore, we could proceed in our endeavor. The project certainly had a learning curve since it was a novel technique. We have overcome all the major obstacles in achieving good functional results to a point where we feel confident to include other institutions and a larger cohort of patients.

Although our experience has not been uniformly satisfactory, sufficient success was achieved. Use of the MWA procedure should be continued, as well as further evaluated and improved.

## Ethics Statement

This study was carried out in accordance with the recommendations of Tangdu Hospital Internal Review Board with written informed consent from all subjects. All subjects gave written informed consent in accordance with the Declaration of Helsinki. The protocol was approved by the Tangdu Hospital Internal Review Board.

## Author Contributions

Q-YF designed the study, performed the surgeries, analyzed the data and wrote the manuscript. All authors performed the surgeries and analyzed the data.

### Conflict of Interest Statement

The authors declare that the research was conducted in the absence of any commercial or financial relationships that could be construed as a potential conflict of interest.
